# Two Main Cancer Biomarkers as Molecular Targets of Binase Antitumor Activity

**DOI:** 10.1155/2024/8159893

**Published:** 2024-02-12

**Authors:** Elena Dudkina, Vera Ulyanova, Violetta Asmandiyarova, Valentina Vershinina, Olga Ilinskaya

**Affiliations:** Department of Microbiology, Institute of Fundamental Medicine and Biology, Kazan (Volga Region) Federal University, Kazan 420008, Russia

## Abstract

Cancer is frequently coupled with the disturbance of key signaling pathways. Aberrant activation of the mitogen-activated protein kinase (MAPK) signaling cascade, occurring in over 85% of cancers, is mainly caused by the genetic alterations of its main components—oncogenes EGFR and RAS, and plays a crucial role in cell fate. The importance of EGFR and RAS proteins in a variety of tumors suggests that they would be good therapeutic targets, but at present, no effective targeted therapy against these two oncogenes has been proven. Here, we show that ribonuclease from *Bacillus pumilus* (binase) inhibits MAPK signaling through direct interaction with EGFR and RAS proteins. This effect contributes to the antitumor potential of binase along with its enzymatic activity. Multitargeticity of binase prevents the development of drug resistance, which is considered a major obstacle to effective anticancer treatment.

## 1. Introduction

Mitogen-activated protein kinase (MAPK) cascade combines key signaling pathways that regulate a wide variety of basic cellular processes, including proliferation, differentiation, migration, cell survival, and apoptosis [1]. The MAPK cascade is activated through receptor tyrosine kinases, G protein-coupled receptors, and integrins that perceive numerous signals, such as stimuli from growth and stress factors. MAPK signaling disturbance can be induced via multiple mechanisms, with receptor overexpression and pathway component mutations being the most common ones. In such cases, the MAPK cascade may function even in the absence of appropriate stimuli constitutively activating downstream effectors. Dysregulation of the MAPK cascade may lead to uncontrolled cell division, loss of cell cycle control, and insensitivity to apoptosis induction resulting in the formation of malignant tumors, their metastasis, and resistance to anticancer drugs [2].

Several MAPK signaling pathways have been identified, including extracellular signal-regulated kinase (ERK), c-Jun N-terminal kinase (JNK), and p38 MAPK. Among them, the Ras/Raf/MEK/ERK pathway is the most important one playing a crucial role in cell proliferation and differentiation, while the two other pathways are related to stress response and apoptosis [3]. The alterations in the Ras/Raf/MEK/ERK pathway are a major oncogenic trigger for the development of most cancer types [4]. Most frequently, these aberrations involve disturbances in the membrane epidermal growth factor receptor (EGFR) [5] and the signal-transducing protein RAS [6], making the altered proteins attractive targets for anticancer therapy.

EGFR is a transmembrane protein belonging to the ErbB/HER receptor tyrosine kinase family. Mutations in the EGFR gene and its amplification are associated with colorectal, head and neck, non-small cell lung, genitourinary, and breast cancers [7]. Currently, distinct approaches for EGFR-targeted therapy are available, including EGFR monoclonal antibodies and tyrosine kinase inhibitors [8, 9]. Despite significant progress in the development of EGFR-targeted drugs, one of the limiting factors of the effective therapy is the mutational status of the downstream RAS protein. However, even in the case of wild-type RAS, the effectiveness of the therapy decreases over time, the treatments lead to primary and acquired drug resistance via reactivation of the pathway, and only a few patients have a lasting response to currently available medication [10].

RAS is an effector molecule responsible for signal transduction from ligand-bound EGFR to the nucleus. Mutations in RAS (mainly KRAS) leading to its persistent activation are the most common mutations in cancer, appearing in nearly 30% of all cancer types [11]. Besides, mutated RAS is recognized as a strong predictor of resistance to EGFR-targeted monoclonal antibodies. Therefore, therapeutic inhibition of oncogenic RAS is of great clinical importance. Multiple agents are being developed to target RAS in several cancer types. The most promising agent is a farnesyltransferase inhibitor which affects RAS posttranslational modification and integration into the membrane [12]. However, their application in the clinic was largely disappointing. Over the years, many studies have tried to find approaches and strategies to directly affect KRAS. The first drug AMG510 (sotorasib) directly targeting mutant KRAS (G12C) was approved by the FDA for clinical usage in 2021. Currently, some KRAS-targeted drugs are being tested in late-stage clinical trials. Despite a definite breakthrough in the development of KRAS inhibitors, resistance to sotorasib is increasingly common [13].

Previously, we have shown that binase, a cytotoxic ribonuclease from *Bacillus pumilus*, directly interacts with wild-type KRAS protein in MLE-12 cells, which leads to the inhibition of the MAPK/ERK pathway and induction of apoptosis in tumor cells [14]. Moreover, binase selectively inhibits the growth of tumor cells expressing KIT, AML/ETO, FLT3, E6, and E7 oncogenes [15–17] and suppresses the migration of cancer cells [18]. However, the antitumor potential of binase is mediated not only by the interaction with oncogenes but also involves the cationic nature of the enzyme allowing its predominant interaction with the cancer cells' membranes, as well as the suppression of K_Ca_ channels and RNA cleavage [19]. RNA hydrolysis by binase leads to a decrease in protein biosynthesis and a generation of regulatory RNA molecules, inducing cell apoptosis [20]. Due to the enzymatic nature of binase, cancer cells do not develop any resistance to it.

In this study, we evaluate the effect of binase on the components of the MAPK cascade and assess the ability of the ribonuclease to interact with its main components, EGFR and RAS proteins, which represent promising targets for anticancer therapy.

## 2. Materials and Methods

### 2.1. Cell Cultures

Human alveolar adenocarcinoma A549 cells and breast cancer BT-20 cells were obtained from the American Type Culture Association (Rockville, Maryland, USA). KRAS-transformed derivatives of rat ovarian epithelial cells ROSE 199 A2/5 were provided by the Cell Culture Collection of the Institute of Cytology of Russian Academy of Science (St. Petersburg, Russia).

Cells were maintained at 37°C and 5% CO_2_ in a humidified atmosphere using Eagle's Minimum Essential Medium (EMEM; PanEco, Russia) for BT-20 cells, RPMI 1640 medium (EMEM; PanEco, Russia) for A549 cells, and *α*-MEM medium (EMEM; PanEco, Russia) in the case of ROSE 199 A2/5 cells. All media were supplemented with 10% fetal bovine serum (FBS; HyClone, United States), 2 mM glutamine, and antibiotics (penicillin and streptomycin, 100 U/mL each).

### 2.2. Binase and Anti-Binase Antibodies

Bacterial ribonuclease binase (12.3 kDa, pI 9.5) was purified as described earlier [21, 22]. Polyclonal rabbit anti-binase antibodies were isolated and purified from the sera of immunized animals as described in [23].

### 2.3. Cell Proliferation Assay

The cell viability was assessed by a standard MTT [3-(4,5-dimethylthiazol-2-yl)-2,5-diphenyltetrazolium bromide] (Merck, Darmstadt, Germany) assay, which detects dehydrogenase activity in viable cells. Cells were seeded (A549-5000 cells/well, BT-20–18000 cells/well) in a 96-well plate and cultured for 24 h. The following day, cells were treated with binase (300 *μ*g/mL), cetuximab (CTX; 100 *μ*g/mL) (Merck, Darmstadt, Germany), and zoledronic acid (ZA; 100 *μ*g/mL) (Pharmidea, Olaine, Latvia) for 24-48 h in various combinations. Cells were incubated with binase, cetuximab, and zoledronic acid alone or in combinations for 48 h. The viability of untreated cells was taken as 100%. In the case of sequential treatment, cells were firstly pretreated with binase for 24 h and then treated with cetuximab for an additional 48 h and vice versa. A similar sequential exposure was reproduced by replacing cetuximab with zoledronic acid. The viability of cells treated by the first agent alone for 24 h followed by media replacement and cultivation for another 48 h was taken for 100%.

For experiments involving EGF treatment, cells were serum-starved for 24 h before stimulation with 100 ng/mL recombinant human EGF (Alomone, Jerusalem, Israel) for 24 h and incubated with binase for an additional 48 h.

At the end of incubation, the culture medium was aspirated and MTT reagent was added to a final concentration of 0.5 mg/mL. Samples were incubated for 2 h at 37°C and 5% CO_2_ in a humidified atmosphere. MTT solution was then removed, and cells were lysed with 100 *μ*L of DMSO. Absorbance was measured at 570 nm using a microplate reader (xMark, Bio-Rad, USA).

### 2.4. Coimmunoprecipitation Assay

The ability of binase to interact with EGFR and RAS proteins was assessed by Abcam's Immunoprecipitation Kit (ab206996; Abcam, USA). BT-20 cells and KRAS-transformed ROSE 199 A2/5 cells were seeded by 150000 cells/well in a 6-well plate and grown for 24 h (90% confluence). Afterwards, cells were lysed with nondenaturing lysis buffer (Abcam, USA) containing a protease inhibitor cocktail (Invitrogen, USA). Total protein content was quantified with Pierce Coomassie (Bradford) Protein Assay Kit (Thermo Scientific, USA).

For immunoprecipitation, 20 *μ*g of binase protein was incubated with 1000 *μ*g of each cell protein lysate for 15 min at 37°C on a shaker with 200 rpm and kept on ice. Then, 1 *μ*g primary mouse monoclonal antibodies against EGFR (sc-120; Santa Cruz, USA) or 1 *μ*g primary mouse polyclonal anti-IgG antibodies as a control (sc-2025; Santa Cruz, USA) were added to BT-20 cell protein lysates and incubated at 4°C overnight. In the case of ROSE 199 A2/5, 1 *μ*g primary mouse monoclonal antibodies against pan Ras (sc-166691; Santa Cruz, USA) instead of anti-EGFR antibodies were added. The immunocomplexes were precipitated with 25 *μ*L prewashed Protein A/G Sepharose beads for 1 h at 4°C. Beads were collected and loaded on 7% or 16% SDS-PAGE for western blot analysis as described under 4.4.

### 2.5. Western Blotting

The precipitated immune complexes were analyzed by western blotting. After SDS-PAGE, proteins were transferred to the PVDF membrane, which was blocked for 1 h with 5% (*w*/*v*) nonfat powdered milk in phosphate-buffered saline (PBS) with 0.2% Tween and stained with anti-binase antibodies [23] at 1 : 500 at 4°C overnight. EGFR and RAS proteins in cell lysates were detected using anti-pan Ras (1 : 100) (sc-166691; Santa Cruz, USA) and anti-EGFR (1 : 200) antibodies (sc-120; Santa Cruz, USA). The primary antibodies were visualized using horseradish peroxidase-conjugated goat anti-rabbit (G-21234; Invitrogen,Carlsbad, CA, USA) at 1 : 10000 and anti-mouse IgG (H + L) antibodies (ab205719; Invitrogen, Carlsbad, CA, USA) at 1 : 5000 for 1 h at room temperature. Proteins were visualized using an ECL chemiluminescent substrate (Pierce, USA) on a ChemiDocXRS+ gel documenter (Bio-Rad, USA).

### 2.6. Cell Signaling Multiplex Assay

Changes in the levels of phosphorylated proteins in the MAPK signaling pathway induced by binase treatment were assessed by phospholuminex assay using 10-plex MAPK/SAPK Signaling Magnetic Bead Panel (Milliplex, Millipore Corporation) which measures phosphorylated ERK1/2 MAPK (Thr185/Tyr187), STAT1 (Tyr701), JNK (Thr183/Tyr185), MEK1 (Ser217/221), MSK1 (Ser212), ATF2 (Thr71), p53 (Ser15), HSP27 (Ser78), c-Jun (Ser73), and p38 MAPK (Thr180/Tyr182). To normalize protein expression, a single-plex Luminex analyte measuring the expression of the housekeeping protein GAPDH (46-667MAG) was added to the assay.

A549 (25000 cells/well) and BT-20 (150000 cells/well) cells were seeded in a 24-well plate and cultured for 24 h. Stimulation of the MAPK signaling pathway was carried out by 50 ng/mL EGF for 30 min after cells were depleted from serum for another 24 h. Binase (300 mg/mL) treatment was performed for 30 and 90 min. Then, cells were lysed with a kit-specific lysis buffer containing a cocktail of protease/phosphatase inhibitors (Thermo Fisher Scientific, USA). Total protein concentrations of the lysates were measured using the Bradford assay (Bio-Rad, USA); 25 *μ*g of total protein was added to each well. Samples were mixed with antibody capture magnetic beads in assay buffer, loaded into a 96-well microplate, and treated according to the manufacturer's instructions.

Data acquisition was carried out using a MAGPIX instrument and analyzed with Milliplex Analyst Software (Millipore, Billerica, MA). Data were expressed as median fluorescent intensity (MFI) of the immunoreactive multiplex beads and normalized to the MFI value of GAPDH from the same sample. Normalized MFI was then standardized to the value of the control sample that was arbitrarily set to one. Data were presented as the fold change of fluorescence intensity of binase-treated cells compared to the binase-untreated cells.

### 2.7. Immunofluorescence Microscopy

A549 cells (25000 cells/well) and BT-20 cells (150000 cells/well) were seeded in 4-well chamber slides and incubated for 24 h in 800 mL of 10% fetal bovine serum containing RPMI or DMEM, respectively. After that, cells were treated with binase (100 *μ*g/mL) for 1 min, 15 min, 60 min, 24 h, and 48 h. After incubation, the medium was removed, and the cells were washed three times with PBS. Then, cells were fixed in a 4% paraformaldehyde solution for 15 min and permeabilized in 0.1% Triton-X100 solution in PBS for 10 min. Binase-treated BT-20 cells were incubated overnight with anti-binase (1 : 25) [23] and anti-EGFR (1 : 50) antibodies (sc-120; Santa Cruz, USA) at 4°C. For A549 cells, anti-binase (1 : 25) and anti-pan RAS (1 : 50) antibodies (sc-166691; Santa Cruz, USA) were used. Next, cells were washed in PBS containing 0.1% Tween and incubated for 1 h at room temperature with secondary goat anti-mouse Alexa Fluor 647-conjugated (ab130782; Abcam, USA) (0.1 *μ*g/106 cells) and goat anti-rabbit Alexa Fluor 555-conjugated antibodies (ab150118; Abcam, USA) at 1 : 200. Finally, cells were counterstained with 40,6-diamidino-2-phenylindole (DAPI) for nuclei visualization. Confocal laser scanning microscope observations were conducted using an Olympus IX83 inverted microscope (Olympus Corporation, Japan) supplemented with a STEDYCON ultrawide extension platform (Abberior Instruments, Göttingen, Germany) at the 405 nm excitation wavelength of the laser for DAPI, 647 nm laser for Alexa Fluor 647, and 488 nm laser for Alexa Fluor 555.

### 2.8. Modelling

The modelling of protein-protein interaction between binase (PDB 1buj, chain A) and EGFR in active (PDB 1ivo, chain A) and inactive (PDB 1nql, chain A) forms was performed by the direct method through a search for structures with minimum Gibbs free energy using the ClusPro server in receptor-ligand mode [24]. The algorithm classifies the predicted models into clusters based on the forces involved in the protein complex formation (electrostatic, van der Waals and electrostatic, hydrophobic, or their balance). The structure with the lowest free energy from the cluster with the largest number of members was chosen. Jmol: an open-source Java viewer for chemical structures in 3D (http://www.jmol.org/ (accessed on 27 January 2023)) was used to visualize 3D structures.

### 2.9. Statistical Analysis

The experiments were carried out in biological triplicates (i.e., newly prepared cultures and medium) with three independent repeats in each one. Statistical tests and graphical outputs were generated with GraphPad Prism 8 software (GraphPad Software, San Diego, CA, USA). All data are represented as *mean* ± *standard* deviation of the mean (SD). The significance of differences between the means of the two groups was assessed by Student's *t*-test. Multiple group comparisons were performed by one-way analysis of variance (ANOVA) with Tukey's post hoc testing. Significant differences were signified as follows: ^∗^*p* < 0.05, ^∗∗^*p* < 0.01, and ^∗∗∗^*p* < 0.001.

## 3. Results

### 3.1. Binase Differently Modulates the MAPK Signaling in A549 and BT-20 Cells

To evaluate the effect of binase on MAPK signaling, we applied a comprehensive immunochemical approach based on a multiplex bead assay for 10 phosphorylated proteins involved in MAPK signaling, namely, ERK, JNK, p38, HSP27, c-Jun, p53, STAT1, ATF2, MSK1, and MEK1. The analysis was performed in BT-20 and A549 cells, which are characterized by constitutively hyperactivated MAPK signaling due to amplification in EGFR and mutation in KRAS, respectively. Cells were treated with binase (300 *μ*g/mL) for 90 min in FBS-containing media.

**Figure 1:**
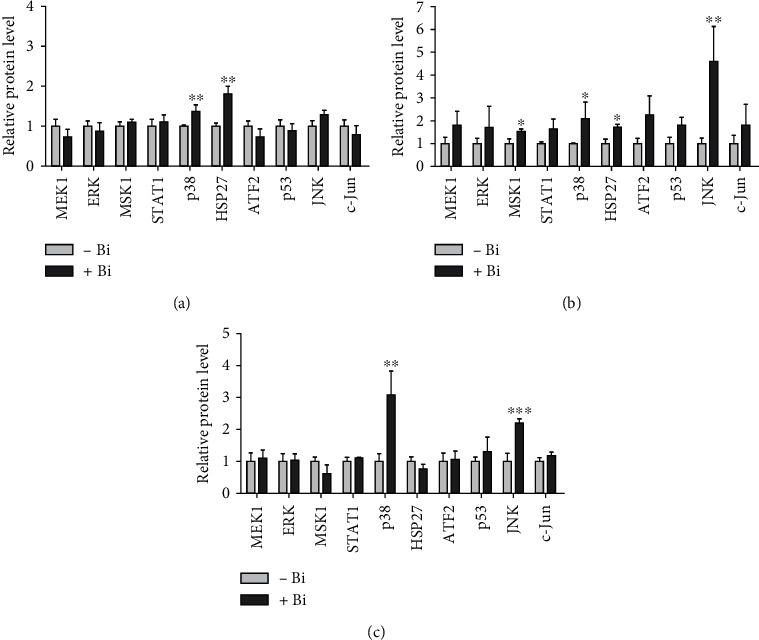
The effect of binase on the MAPK phosphorylation in BT-20 and A549 cells. The (a) BT-20 and (b) A549 cells were treated with binase (300 *μ*g/mL) for 90 min on FBS-containing media. (c) The A549 cells were treated with binase (300 *μ*g/mL) for 90 min on a serum-starved medium. Data represent the fold change of fluorescence intensity of binase-treated cells as compared to the binase-untreated cells. Significant differences are indicated as ^∗^*p* < 0.05, ^∗∗^*p* < 0.01, and ^∗∗∗^*p* < 0.001.

We found that binase increased the phosphorylation level of stress-related p38 and HSP27 proteins in BT-20 cells (Figure 1(a)). In A549 cells, all MAPK proteins, especially JNK, were activated by binase treatment (Figure 1(b)). To exclude the influence of culture media components on the MAPK cascade activation, we repeated the experiment for A549 cells by cultivating them in the serum-free medium. Under these conditions, only kinases p38 and JNK were activated (Figure 1(c)). Therefore, A549 cells perceive binase as a stress factor by activating stress-related pathways of the MAPK cascade.

### 3.2. Binase Colocalizes with EGFR and RAS Proteins

MAPK cascade plays a key role in tumorigenesis wherein genetic alterations in KRAS and EGFR genes are leading trigger factors of cancer. Earlier, it was demonstrated that binase directly binds to wild-type KRAS protein [14]. Considering the binase ability to affect MAPK cascade which is activated by stimuli perceived mainly by EGF receptor, we analyzed the ability of binase to interact with EGFR in BT-20 cells and mutated KRAS in A549 cells. We treated both cell lines with binase (100 *μ*g/mL) for 1 min, 15 min, 60 min, 24 h, and 48 h and performed immunofluorescence analysis.

**Figure 2:**
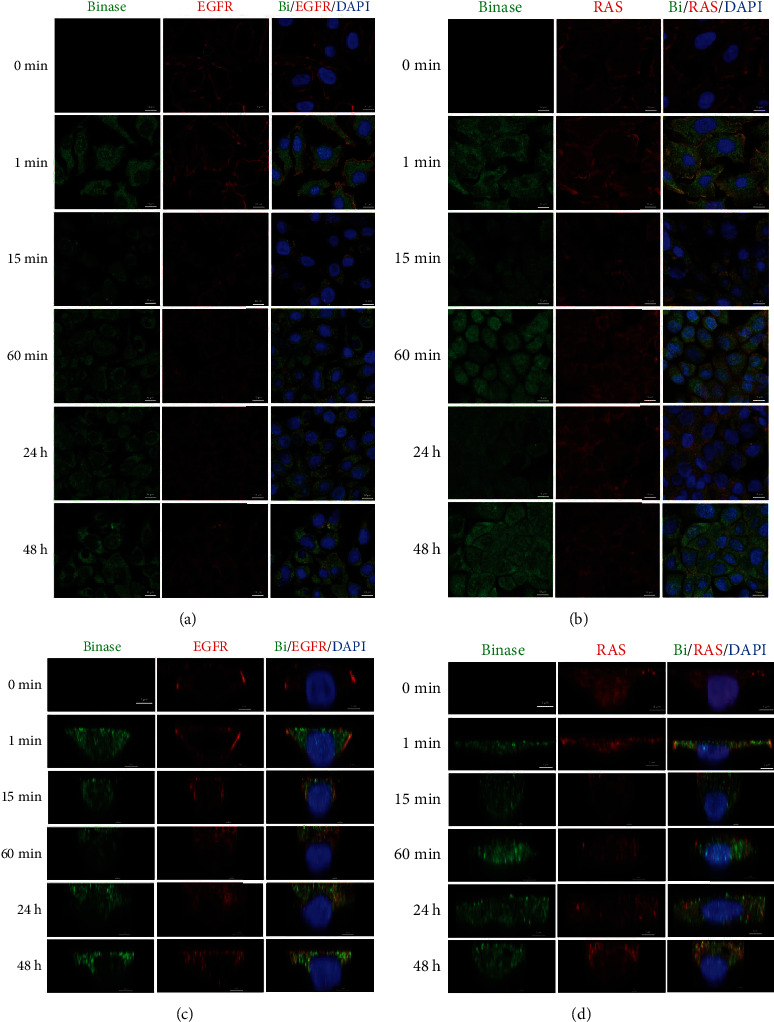
The immunofluorescent colocalization of binase with EGFR and RAS proteins. The colocalization of binase with EGFR protein was assessed in BT-20 cells (a, c) and with RAS protein in A549 cells (b, d). Panels (a) and (b) represent the overall image of the cells while panels (c) and (d) show the cross-sections of the individual cells. In the assay, cells were grown on glass coverslips, treated with binase (100 *μ*g/mL) for 1 min, 15 min, 60 min, 24 h, and 48 h or left untreated (control), and fixed and stained with the primary mouse monoclonal anti-EGFR (sc-120) or mouse anti-pan Ras (sc-166691) and rabbit anti-binase antibodies followed by the secondary antibodies conjugated to Alexa Fluor 647 (for EGFR and pan Ras, red) and Alexa Fluor 555 (for binase, green). The cell nuclei were stained with DAPI.

Binase rapidly penetrated into the cells since it was detected in large amounts already after 1 min of incubation; after 15 min, binase was diffusely distributed in the cytosol and nucleus of both cell lines; from 60 min and up to 48 h, it was vizualized mostly in the cytosol of BT-20 cells (Figure 2(a)), while in A549 cells, it accumulated in the nucleus and near it (Figure 2(b)). EGFR and RAS proteins resided predominantly in the cell membrane of the untreated cells as evidenced by a strong peripheral staining (Figures 2(a) and 2(b)). Binase treatment induced their translocation from the plasma membrane to the perinuclear space. After 15 min, the bulk of EGFR protein was localized in cytoplasmic compartments, presumably in endosomes; the recycling of the EGF receptor to the plasma membrane was not observed.

The protein distribution analysis revealed that binase colocalized both temporally and spatially with EGFR and RAS proteins inside BT-20 and A549 cells (Figures 2(c) and 2(d)). After 1 min treatment, the fluorescent signal from binase overlapped either with EGFR or RAS proteins on the cell plasma membrane. Then, up to 24 h binase and RAS cooccured near the nucleus; by 48 h, the signal decreased. After strong initial colocalization with binase on the plasma membrane, EGFR protein internalized into the cell since its membrane fluorescence decreased. The interaction of binase with EGFR was detected at perinuclear regions up to 48 h.

### 3.3. Binase Directly Binds EGFR and RAS Proteins

**Figure 3:**
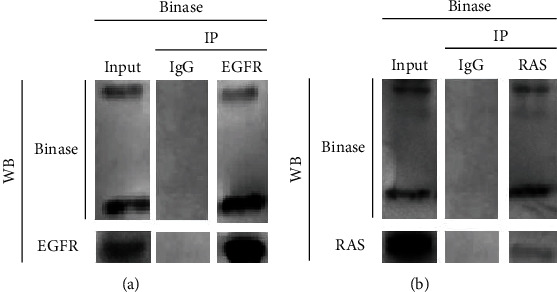
The protein-protein interaction of binase with EGFR and RAS proteins. (a) EGFR was immunoprecipitated from the whole cell protein extracts of BT-20 cells treated with binase (20 *μ*g/mg cell protein extract) using either IgG (control) or anti-EGFR antibodies. (b) RAS was immunoprecipitated from the whole cell protein extracts of ROSE 199 A2/5 cells treated with binase (20 *μ*g/mg cell protein extract) using either immunoglobulin G (IgG, control) or anti-pan Ras antibodies.

To confirm the direct protein-protein interaction of EGFR and mutated KRAS proteins with binase, we performed a coimmunoprecipitation (co-IP) assay. According to the gene expression profiles, two cancer cell lines were chosen: BT-20 overexpressing EGFR with wild-type KRAS and ROSE 199 A2/5 bearing C12V mutation in KRAS with wild-type EGFR. Cell line ROSE 199 A2/5 was chosen for this experiment instead of the A549 cell line because of the more intensive signal from the RAS protein. Initially, we detected the EGFR and RAS proteins in these cell lines using western blot analysis. Furthermore, EGFR and RAS proteins were immunoprecipitated from whole cell lysates of BT-20 (Figure 3(a)) and ROSE 199 A2/5 cells (Figure 3(b)), respectively, preincubated with binase (20 *μ*g) using anti-EGFR and anti-pan Ras antibodies. Adsorption of interacting protein complexes from cell lysates using anti-binase antibodies was unsuccessful. We hypothesized that binase interaction with EGFR and RAS proteins interferes with the binding of anti-binase antibodies to binase. The analysis of the isolated protein complexes using anti-binase antibodies showed the presence of binase in the immunoprecipitated samples (Figure 3). Therefore, binase coimmunoprecipitated endogenous EGFR and mutated RAS proteins demonstrating a direct interaction with them. Binase was detected as two bands of 12 kDa and 25 kDa reflecting its dimeric nature. The obtained results confirmed previously performed molecular modelling of binase interaction with RAS protein [14], demonstrating the ability of the ribonuclease to bind both mutant and wild-type KRAS proteins.

### 3.4. Binase Binds to EGFR at the Same Region as EGF

**Figure 4:**
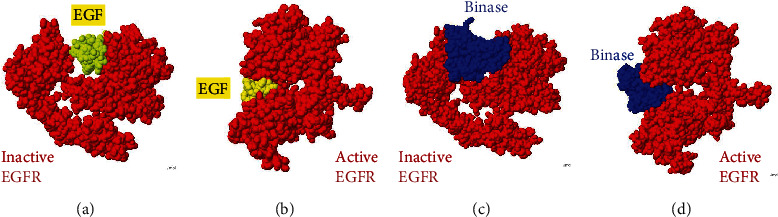
The molecular modelling of the interaction between binase and EGFR. The structure of the extracellular domain of human EGFR (red) is shown in the (a) inactive (PDB 1nql) and (b) active (PDB 1ivo) complexes with EGF (yellow). Only one monomer of the active EGFR conformation is presented. The models of binase (blue) interaction with EGFR (red) in the (c) inactive and (d) active conformations extracted from the PDB structures 1nqlA and 1ivoA, respectively.

To determine the regions in the EGFR protein which can be involved in the interaction with binase, we applied molecular modelling. In the absence of a ligand, EGFR exists as an inactive monomer. Upon ligand binding, EGFR is activated by dimerization and autophosphorylation, which leads to receptor endocytosis and further signal transduction to downstream proteins [25]. The EGF-EGFR binding interface is formed by subdomains I (residues 1–165) and III (residues 310–481) of the receptor extracellular domain and loops A (residues 6–19) and B (residues 20–31) of EGF. The interaction is mediated by hydrophobic and electrostatic forces. The modelling showed that the extracellular domain of EGFR binds binase in the same area as its natural ligand EGF, both in the active and inactive conformations (Figure 4).

**Figure 5:**
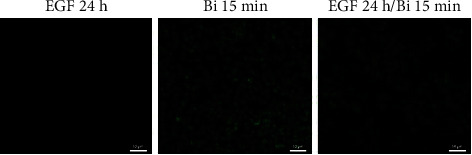
The immunofluorescent analysis of binase internalization into BT-20 cells. The cells were grown on glass coverslips, treated with EGF (100 ng/mL) for 24 h and/or binase (100 *μ*g/mL) for 15 min, and fixed and stained with the primary rabbit anti-binase antibodies followed by the secondary antibodies conjugated to Alexa Fluor 555 (binase, green). The scale bar is 50 *μ*m.

To verify the possible competition of binase and EGF for the same binding site on the EGFR surface, we examined the internalization of binase into cells after their treatment with EGF (100 ng/mL) which usually induces EGFR trafficking from the cell surface [26]. We detected that the amount of internalized binase after EGF treatment decreased (Figure 5). These results indicated that binase utilized the EGF receptor for its cellular internalization. Besides, cell treatment with EGF led to the elimination of binase cytotoxicity. Binase (300 *μ*g/mL) inhibited the growth of BT-20 cells by ~25% after 48 h of incubation, while the cells' pretreatment with EGF (100 ng/mL) for 24 h made them unsensitive to the binase cytotoxic action (data not shown). Therefore, we supposed that binase can compete with EGF for EGFR binding and modulate EGFR-related signal transduction.

### 3.5. Binase Interferes with EGF Signaling

**Figure 6:**
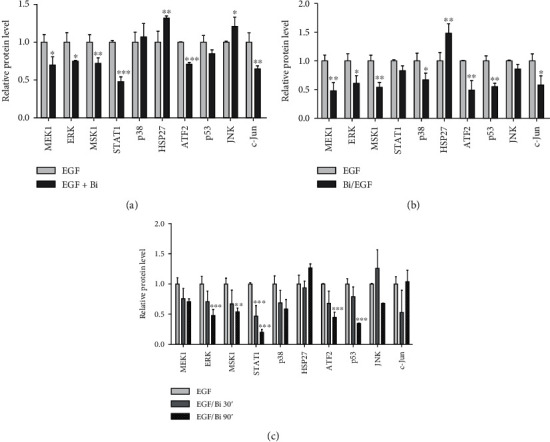
The effect of binase on EGF-stimulated MAPK phosphorylation in BT-20 cells. (a) Cells were stimulated with EGF alone or in combination with binase for 30 min on a serum-starved medium. (b) Cells were stimulated with EGF for 30 min on a serum-starved medium with and without their pretreatment with binase for 30 min. (c) Cells were stimulated with EGF for 30 min on serum-starved medium followed by treatment with binase for 30 and 90 min. Data represent the fold change of fluorescence intensity of binase-treated cells as compared to the binase-untreated cells. Significant differences are indicated as ^∗^*p* < 0.05, ^∗∗^*p* < 0.01, and ^∗∗∗^*p* < 0.001.

To evaluate whether binase can compete with EGF for EGFR binding, we analyzed the changes in EGF-induced signal transduction by the MAPK cascade. Using the multiplex assay, we assessed the levels of phosphorylated MAPK proteins in BT-20 cells overexpressing EGFR. Combined cell treatment with EGF and binase for 30 min reduced MAPK/ERK phosphorylation in comparison with the EGF treatment alone (Figure 6(a)). The lowered level of phosphorylated transcription factors ATF2, c-Jun, STAT1, and MSK1 as well as MEK1 and ERK kinases was observed. Cell pretreatment with binase for 30 min prior to stimulation by EGF decreased the EGF's ability to activate the MAPK cascade, which was expressed by the reduced phosphorylation of most proteins, except for HSP27 (Figure 6(b)). Therefore, upon EGFR binding at the same region as EGF, binase interfered with EGF signaling.

To investigate the ability of binase to inhibit signal transmission in the activated MAPK cascade, we incubated the EGF-treated BT-20 cells with binase for 30 and 90 min. Upon binase treatment, the phosphorylation of most components of the MAPK cascade except for HSP27 and c-Jun decreased over time (Figure 6(c)), suggesting that binase inhibited the EGFR-activated MAPK cascade.

### 3.6. Binase Potentiates the Cytotoxic Action of Anti-EGFR and Anti-RAS Agents

**Figure 7:**
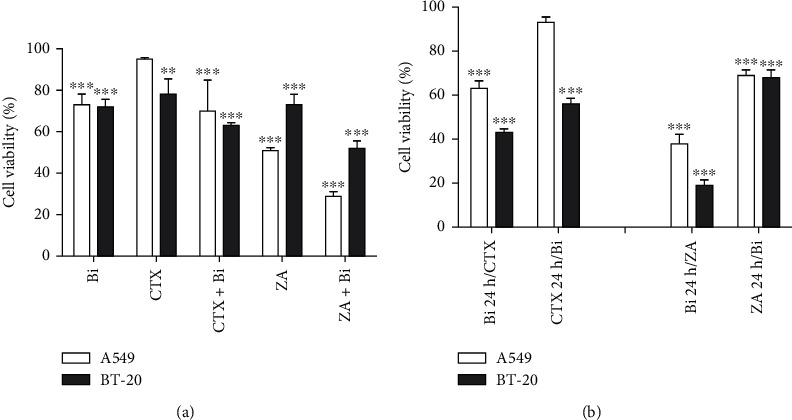
The effect of binase, cetuximab, and zoledronic acid and their combinations on the cell viability of A549 and BT-20 cells as measured via the MTT assay. (a) The cells were incubated with binase (300 *μ*g/mL), cetuximab (100 *μ*g/mL), and zoledronic acid (100 *μ*g/mL) or their combinations for 48 h. The viability of the untreated cells was taken for 100%. (b) The cells were incubated with the first agent for 24 h followed by the incubation with the second drug for 48 h at the abovementioned concentrations. The viability of the cells treated by the first agent alone for 24 h followed by the media replacement and cultivation for another 48 h was taken for 100%. Significant differences are indicated as ^∗∗^*p* < 0.01 and ^∗∗∗^*p* < 0.001.

To confirm that EGFR and RAS are targets of binase cytotoxic action, we evaluated the antiproliferative effect of binase in the presence of the anti-EGFR monoclonal antibodies cetuximab and zoledronic acid indirectly targeting RAS by inhibition of its prenylation and membrane integration [27]. Sensitive to these drugs BT-20 and A549 cell lines were treated with 300 *μ*g/mL binase, 100 *μ*g/mL cetuximab, and 100 *μ*g/mL zoledronic acid for 48 h in different combinations. Binase treatment decreased the growth of both tumor cell lines by approximately 27%, while cetuximab declined the cell viability of BT-20 cells by 22% and A549 cells by 5% (Figure 7(a)). The metabolic activity of BT-20 and A549 cells after 48 h incubation with zoledronic acid lowered by 27% and 49%, respectively (Figure 7(a)). An additive interaction effect between binase and either cetuximab or zoledronic acid was estimated by comparing the viability of the cells treated by cetuximab/zoledronic acid and binase simultaneously to the cells treated by each agent alone. Binase in combination with cetuximab decreased the viability of cetuximab-sensitive BT-20 cells by 37% and cetuximab-insensitive A549 cells by 30% (Figure 7(a)). In the case of binase combination with zoledronic acid, the cell growth decreased by 48% for BT-20 cells and by 71% for A549 cells (Figure 7(a)). Therefore, the combined effect of binase with zoledronic acid was greater than their individual potencies, meaning that they exerted synergistic action towards cancer cells. In the case of cetuximab, its combined effect with binase was less pronounced.

To evaluate the cytotoxic effects of binase, cetuximab, and zoledronic acid upon sequential drug administration, BT-20 and A549 cell lines were subjected to the following treatment regimens: (1) treatment with binase for 24 h followed by cetuximab treatment for 48 h and (2) the treatment with cetuximab for 24 h followed by binase treatment for 48 h. The same procedure was reproduced using zoledronic acid instead of cetuximab. The pretreatment of A549 cells with cetuximab decreased the cytotoxic effect of binase, while pretreatment with binase enhanced the antiproliferative effect of cetuximab. EGFR blocking by cetuximab led to the complete loss of cytotoxicity of binase, indicating that EGFR is its primary target. For BT-20 cells, sensitive to both cetuximab and binase, such an obvious effect was not observed. Binase and cetuximab strengthened each other's antitumor effect.

The pretreatment of BT-20 and A549 cells with zoledronic acid followed by binase treatment resulted in a decrease in cell viability by 30% (Figure 7(b)). Preadded zoledronic acid inhibited RAS translocation to the membrane, eliminating it from the signal transduction network [27] and making it inaccessible to binase action. The subsequent addition of binase, which can target both EGFR and RAS, insignificantly contributed to the cytotoxicity because blocking of upstream EGFR is not effective in the case of nonfunctional RAS. Conversely, cell pretreatment with binase followed by zoledronic acid decreased their proliferation drastically (Figure 7(b)), which can be explained by the simultaneous inhibition of two main proteins of the MAPK signaling pathway, EGFR and RAS, both by zoledronic acid and binase. The obtained results suggested that EGFR and RAS are targets of the binase antitumor effect, with the EGF receptor being a more preferable one.

## 4. Discussion

Tumorigenesis is often associated with atypical forms of cell regulatory networks, especially with their hyperexpression. Aberrant EGFR signaling, affecting the MAPK cascade, contributes to tumor invasion, metastasis, and progression. The main signal transducer of the MAPK cascade is the KRAS protein. Currently, EGFR and RAS proteins, which are both aberrantly activated in a wide range of human cancers, are regarded as central targets for anticancer drug development [28, 29]. Despite the availability of targeted drugs, anticancer therapy is limited in the clinic because of drug resistance emerging due to mutational changes in tumor cells during tumor progression. Therefore, compounds affecting different targets regardless of their mutational status are considered the most attractive ones. Among them, binase, the ribonuclease from *Bacillus pumilus*, that possesses selective antitumor activity affecting various intracellular targets, is a promising agent [16].

In this study, we analyzed the effect of binase on the key components of the MAPK cascade from the surface receptor EGFR to cellular kinases and transcription factors. We showed that binase inhibits MAPK signaling by acting on EGFR and RAS proteins through direct interaction with them. Based on our results, we suppose that binase interaction with cancer cells starts from the binding to the EGF receptor. The binase-EGFR protein complex was detected on the cell membrane of the triple-negative BT-20 breast cancer cell line overexpressing EGFR 1 min later of cell treatment with binase (Figures 2(a) and 2(b)). The direct protein-protein interaction was confirmed by the coimmunoprecipitation assay (Figure 3). Using molecular modelling, we established that binase binds to EGFR in the same region as its natural ligand EGF (Figure 4). Experimentally, we showed that binase and EGF compete for the EGFR binding. Cell pretreatment with binase reduced the EGF stimulation of the MAPK cascade (Figure 6). In turn, EGF pretreatment decreased binase internalization and cytotoxicity (Figure 5). Moreover, EGFR inhibition by cetuximab also diminished the antiproliferative effect of binase (Figure 7). The obtained results indicate that the EGF receptor is the direct but not the only target of binase.

Furthermore, the binase-EGFR interaction induces their internalization inside the cells. During 48 h, EGFR localizes in the perinuclear space without membrane recycling, while binase is distributed throughout the cell including the nucleus, and distinct EGFR-binase colocalization signals are detected near the nucleus (Figures 2(a) and 2(c)). Binase interaction with EGFR interferes with its downstream signaling via the MAPK cascade. Using the multiplex assay, we confirmed the decreasing levels of the phosphorylated components constituting ERK, p38, and JNK pathways of the MAPK cascade (Figure 6). This effect can be explained by the inhibition of a component that is common for all three pathways of the MAPK cascade, and this component is the EGF receptor. Binase binding to EGFR impairs its ability to transmit signals leading to the inhibition of the MAPK cascade. The inverse effect was reported for bovine RNase A and human RNase 5 (angiogenin), which upon interaction with EGFR stimulate its signaling and trigger cell oncogenic transformation [30]. The obtained results suggest that EGFR binding contributes to the cytotoxic potential of binase and underlines the importance of blocking the upstream components of the signaling network. However, the efficiency of such an approach is highly dependent on the mutational status of the downstream transducer KRAS [31].

Earlier, binase was found to interact with the wild-type KRAS protein in MLE-12 cells that lead to MAPK/ERK inhibition and induction of cell apoptosis [14]. Here, we showed that binase is also able to bind directly to the mutant RAS (Figures 2(d) and 3). Colocalization of binase and RAS proteins was detected on the membrane of A549 cells bearing KRAS G12S mutation after 1 min of incubation with binase. After 15 min and up to 48 h, binase and RAS are visualized near the nucleus (Figures 2(b) and 2(d)). Binase decreases the viability of A549 cells by 27% (Figure 7). Using zoledronic acid as an inhibitor of RAS-mediated signaling, we showed that binase antiproliferative effect significantly enhances if combined with zoledronic acid (Figure 7). The treatment of A549 cells with both binase and zoledronic acid reduced the metabolic cell activity by 48%, indicating their synergistic effect (Figure 7). This effect raises upon cell preexposure to binase in the sequential order with zoledronic acid, while cell pretreatment with zoledronic acid does not allow binase to fully exert its antitumor potential (Figure 7). These results demonstrate that binase and zoledronic acid act on the same target. Binase can directly interact with RAS protein regardless of its mutational state and block its downstream signaling. However, the antitumor potential of binase is not limited to RAS only and definitely involves additional targets.

Therefore, the inhibition of the MAPK cascade via interaction with EGFR and RAS proteins contributes to the antitumor effect of the ribonuclease binase along with cellular RNA cleavage. Targeting towards EGFR and RAS oncogenes with a single agent was unsuccessful so far. Despite the fact that the combined usage of EGFR- and RAS-targeting drugs in clinical practice enhances the effectiveness of cancer treatment [32], MAPK signaling inhibitors cause cell resistance by activating compensatory feedback loops in tumor cells and tumor microenvironment components [33, 34]. Due to the enzymatic nature and multitargeticity, binase itself lacks the ability to induce drug resistance, which makes it a promising anticancer drug both in a single formulation and in combination with other compounds, such as zoledronic acid and cetuximab.

## Data Availability

The data used to support the findings of this study are available from the corresponding author upon request.
